# Identification and characteristics of combined agrometeorological disasters caused by low temperature in a rice growing region in Liaoning Province, China

**DOI:** 10.1038/s41598-021-89227-y

**Published:** 2021-05-11

**Authors:** Ruipeng Ji, Wenying Yu, Rui Feng, Jinwen Wu, Yushu Zhang

**Affiliations:** 1 Institute of Atmospheric Environment, China Meteorological Administration, Shenyang, 110166 China; 2 Key Laboratory of Agrometeorological Disasters, Liaoning Province, Shenyang, 110166 China

**Keywords:** Natural hazards, Agroecology, Atmospheric science, Climate change

## Abstract

Owing to climate change, agrometeorological disasters are becoming increasingly complex. Here, we analysed the characteristics of combined agrometeorological disaster (CAD) caused by low temperature in annual rice crops in Liaoning Province, China, from 1961 to 2017. We assessed the repeat occurrence of natural disasters on rice production. The results showed that (1) there were six possible CAD scenarios in a rice growing season. These included two scenarios with one disaster in two periods (OD-1, OD-2), three scenarios with two different disasters (TD-1, TD-2, TD-3) and one with multiple disasters (MD-1). Since 1961, the overall occurrence of the six CAD scenarios showed a downward trend. Among the six scenarios, TD-1 had the greatest distribution and occurred most frequently; (2) three possible single agrometeorological disaster (SAD) scenarios may occur during a rice growing season, delayed cold damage (SAD-d), frost damage at only one stage (SAD-f), sterile-type cold damage at one stage (SAD-s). Since 1961, the SAD-d frequency decreased, whereas, since the mid-1980s, the SAD-f frequency increased; (3) SAD and CAD frequencies showed downward trends, with CAD declining more than SAD. The CAD geographical range and frequency were smaller than those of SAD. Rice damage in SAD-f and OD-1 scenarios showed no significant trend, but appeared to have slightly increased. The main agrometeorological disasters affecting rice production in Liaoning Province were delayed cold damage, frost damage or both; (4) a comparison of the rice yield reduction rates of years in which CAD or SAD occurred in more than 50% of stations in Liaoning Province revealed that the yield reduction rates associated with the former were greater than those associated with the latter. CAD had more types, and the occurrences and impacts were more complicated, than for SAD. Compared with SAD, the effects of CAD may be magnified in rice crops, leading to reduced yields.

## Introduction

Rice (*Oryza sativa L.*) is an important crop worldwide and is planted in a variety of environments from 35° S to 53° N and at altitudes of 0 to greater than 2000 m^1^. The Food and Agriculture Organization predicts that the world’s population will grow by 34%, resulting in another 2.3 billion people needing to be fed, by 2050^2^. Rice has become a main food crop in response to population growth^3^, and global rice production must increase by 0.6%–0.9% per year by 2050 to feed approximately half of the world’s population^4^. Potential reductions in rice yields may result from meteorological, disease- and pest- related disasters^5^. Therefore, any fluctuations in rice yield owing to agrometeorological disasters will have a direct effect on world food security. To date, most studies have focused on a single rice-related agrometeorological disaster. For example, Shimono et al.^6, 7^ simulated a low temperature environment (water, air and estimated panicle temperatures) in a 4-year field experiment and found that low temperatures significantly increased rice sterility during the vegetative growth stage. Storms are also a major factor resulting in rice production losses in southwest Japan^5^. Tani^8^ indicated that the yield losses caused by storms were mainly the results of interactions between high wind speeds and heavy rainfalls. In the Yangtze River Basin and Southern China, Xiong et al.^9^ studied the effects of droughts and floods, as well as their reversal, on the physiological characteristics and yield composition of rice at the tillering stage; and found that the yields per plant in 2014 decreased by 29.5%, 11.4%, and 37.7%, respectively, indicating that drought has a greater effect than flooding. Using the A1B emission scenario of the Intergovernmental Panel on Climate Change and the normalized production damage index, Teixeira et al.^10^ simulated the global risk of heat stress and found that rice showed a high level of heat-stress-related damage under basic climatic conditions (1971–2000), especially in South Asia. Mamun et al.^11^ reported that low temperatures inhibit the development of rice microspores, leading to male sterility. Matsui et al.^12^ studied the differences in sterility in nine varieties of rice caused by high temperatures at the flowering stage and concluded that the most sensitive and the most tolerant varieties had their fertility levels reduced to 50% at approximately 37 °C and 40 °C, respectively. For rice grown in China, cold and heat damage caused by low and high temperatures, respectively, are the main meteorological risks^13^. Crops are often affected by a variety of abiotic stresses at the same time, and their responses are unique. They cannot be directly inferred from exposure to a single abiotic stress^14^. Suzuki et al.^15^ found that the root temperature of rice at the seedling stage was an important factor in leaf responses and sensitivity of levels to low-temperature stress. They found that at the seedling stage an air temperature of 10 °C and a root temperature of 25 °C resulted in leaves having significant damage, which would also occur, or even be intensified, in a sunlit environment. Perdomo et al.^16^ assessed the single or combined effects of high-temperature and water-deficit stresses on plant growth, leaf gas exchange and water-use efficiency in the three most important crops in the world—rice, wheat and maize. They found that the accumulated total-crop growth decreased in all the treatments and that high-temperature and water-deficit stresses produce the most damage.

There have been some initial experimental studies and risk assessments of crop agrometeorological disasters. Keles and Öncel^17^ analysed the response of wheat seedlings to the combined stresses of low and high temperatures, as well as the combined stresses of flood, drought and salinity. They found that root and stem elongation decreased significantly under drought and salt-stress conditions. Additionally, low and high temperatures inhibited seedling growth under salt-stress conditions. Rollins et al.^18^ showed that the effects of drought stress on the morphology and growth characteristics of barley were greater than those of heat stress. The latter alone had little effect on barley, whereas the effects of combined drought and heat stress were more serious than the either individual stress. Crops exposed to combined stresses generally have stronger responses, and experience more severe damage, than crops exposed to a single stress. Crops are affected, to different degrees, when multiple disasters occur simultaneously or successively. For instance, when drought and cold disasters occur simultaneously, the effects on crops are greater than the overall effects of the two disasters individually^19^. Combined heat and drought, and combined cold and drought, inflict serious damage to crops^20^. Liu et al.^21^ simulated the effects of low temperatures and drought on the growth and development of maize and found that the combination had a greater effect on yield than either single stress. Wu et al.^22^ indicated that the effects of the combined stress of an enhanced ozone content and ultraviolet B band radiation on soybean biomass and yield were greater than the simple additive effects of the two separate stresses. Wang et al.^23^ reported that the combined stress of soil waterlogging and high temperature significantly decreased wheat quality. Additionally, Wang et al.^24^ found that the effects of a combined high-temperature and drought stress on the physiological parameters of *Bletillastriata* were greater than those of each stress.

In response to global climate change, crop growth and development will be subjected to a greater range and quantity of environmental stresses, which may occur simultaneously and have serious consequences^25^. Previous studies of combined agrometeorological disaster (CAD) have mostly analysed plant stress experimentally, but few studies have been conducted under natural field conditions, especially for rice. Because the CAD occurrence characteristics under natural field conditions are still unclear, conclusions based on the effects of CAD obtained under experimental conditions on plant morphology, physiological traits, and yield may not be applicable for guiding practical agricultural production.

North-east China is an important rice production base. The main agrometeorological disasters that affect rice production are delayed cold damage (DCD), sterile-type cold damage (SCD) and frost damage (FD). There are many methods to identify single agrometeorological disasters, and several indices have been verified by measuring reductions in rice yields. Using the indices developed in previous studies, we identified the occurrences of single agrometeorological disaster (SAD) during rice production in Liaoning Province since 1961. The occurrences of CAD during rice production were then selected, and six scenarios were constructed. The temporal and spatial distributions of the six scenarios were analysed to provide a reference for further research into CAD.

## Materials and methods

### Area

The study site (118° 53′ E–125° 46′ E and 38° 43′ N–43° 26′ N) is in Liaoning Province, in the south of northeast China, with an area 148,000 km^2^. It belongs to a temperate continental monsoon climate, with four distinct seasons. The annual mean temperature is 8.5 °C, the mean annual precipitation is 656 mm and there are 2520 h of sunshine annually. Liaoning Province, a semi-humid region, grows early-maturing rice in a single season, covering an area of 489,120 ha in 2019. The planted varieties are the *japonica* subspecies of cultivated rice in Asia, with 15–17 leaves. The growing season begins with seeding in April and ends with harvesting in September (Table 1). The main agrometeorological disasters are chilling and frost damage caused by abnormally low temperature.

**Table 1:** Main growth stages of rice.

Growing stage	Date range
Seedling stage	21, April–31, May
Booting stage	11, July–31, July
Flowering stage	1, August–31, August
Milk stage	1, September–31, September

### Data

**Figure 1:**
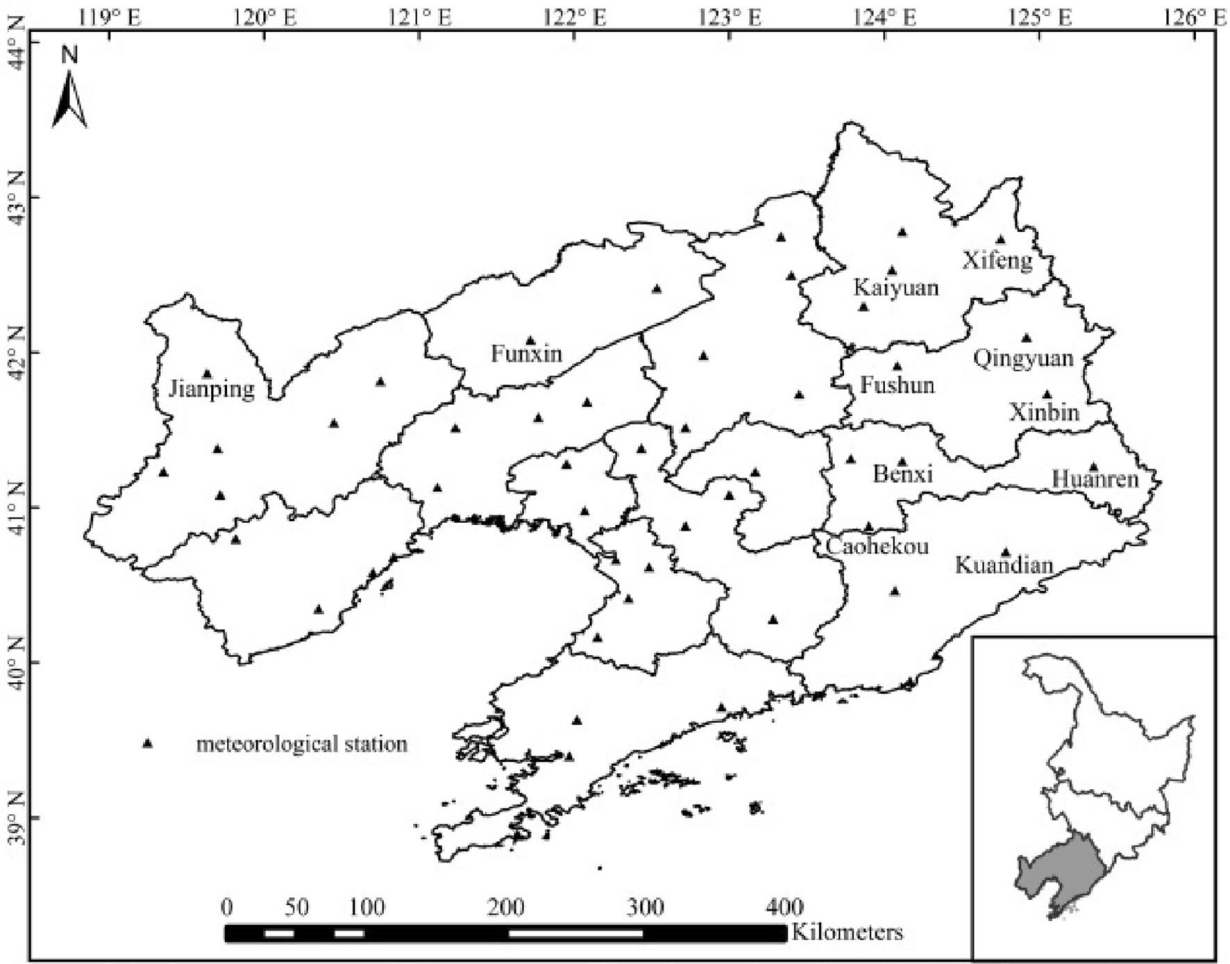
Location of 52 meteorological stations in Liaoning Province. Maps generated in ArcGIS 9.3.

The meteorological and agricultural data, which were from the Liaoning Meteorological Bureau, included diurnal mean temperature, diurnal minimum temperature, and rice development stages from 52 stations in Liaoning Province (Fig. 1) from 1961 to 2017. The rice yield data were obtained from the Liaoning Provincial Bureau of Statistics or the Liaoning Provincial Statistical Yearbook (http://tjj.ln.gov.cn/tjsj/sjcx/ndsj/). The data were used to calculate the agrometeorological disasters affecting rice in Liaoning Province using the methods provided in the next Section. The data were processed and charted in using Excel 2010 (Microsoft Corporation, Redmondm, WA, USA).

### Methods

The following sections show the methods for calculating the indices for DCD, SCD and FD.

#### Delayed cold damage index of rice

The anomaly of the summed mean temperature from May to September ($\Delta T_{5 - 9}$) is commonly used to calculate the DCD of rice in Northeast China^26^, and the calculation formula (Eq. 1) is:

1 $$\Delta {\text{T}}_{5 - 9} = \frac{{{\text{T}}_{5 - 9} }}{{{\overline\sum{T}}_{5 - 9} }}$$

where $\Delta T_{5 - 9}$ is the anomaly of the summed mean temperature from May to September (Table 2); $T_{5 - 9}$ is the summed mean temperature from May to September; $\overline{\sum }T_{5 - 9}$ is a long-term climatic mean value of the summed mean temperature from May to September.

**Table 2:** Delayed cold damage (DCD) index of rice in different heat conditions.

Regional $\overline{\sum }T_{5 - 9}$ [°C]	Light cold damage $\Delta T_{5 - 9}$ [°C]	Moderate cold damage $\Delta T_{5 - 9}$ [°C]	Severe cold damage $\Delta T_{5 - 9}$ [°C]
≤ 83	− 1.0 to − 1.5	− 1.5 to − 2.0	< − 2.0
83.1–88	− 1.1 to − 1.8	− 1.8 to − 2.2	< − 2.2
88.1–93	− 1.3 to − 2.0	− 2.0 to − 2.6	< − 2.6
93.1–98	− 1.7 to − 2.5	− 2.5 to − 3.2	< − 3.2
98.1–103	− 2.4 to − 3.0	− 3.0 to − 3.8	< − 3.8
103.1–108	− 2.8 to − 3.5	− 3.5 to − 4.2	< − 4.2
108.1–115	− 3.6 to − 4.5	− 4.5 to − 5.1	< − 5.1
> 115	− 4.5 to − 5.3	− 5.3 to − 5.9	< − 5.9

#### Sterile-type cold damage index of rice

The rice sterile-type cold damage index (RSCDI) was used to calculate SCD in northeast China^27^, and the calculation formula (Eqs. 3, 4, 5) is:

2 $${\text{RSCDI}} = \frac{{\mathop \sum \nolimits_{{{\text{i}} = 1}}^{{\text{n}}} {\text{ST}}}}{{{\text{SD}}}}$$

3 $${\text{ST}} = \frac{{{\text{S}}\left( {\text{t}} \right)}}{0.54}$$

4 $${\text{S}}\left( {\text{t}} \right) = \frac{{\left( {{\text{T}} - {\text{T}}_{1} } \right)\left( {{\text{T}}_{2} - {\text{T}}} \right)^{{\text{B}}} }}{{\left( {{\text{T}}_{0} - {\text{T}}_{1} } \right)\left( {{\text{T}}_{2} - {\text{T}}_{0} } \right)^{{\text{B}}} }}$$

5 $${\text{B}} = \frac{{{\text{T}}_{2} - {\text{T}}_{0} }}{{{\text{T}}_{0} - {\text{T}}_{1} }}$$

where RSCDI is rice sterile-type cold damage index, range [0, 1); when RSCDI < 1, cold damage may occur; ST is standardized temperature suitability calculated by formula (Eq. 3); $S(t)$ is the temperature suitability of day i calculated by formula (Eq. 4); SD is the duration days when $S(t)$ < 0.54; *n* is the number of days; $T$ is actual mean temperature; $T_{2}$, $T_{1}$, $T_{0}$ represent diurnal maximum temperature, diurnal minimum temperature and the optimum temperature of rice at the booting stage or flowering stage, respectively; B is a coefficient calculated by formula (Eq. 5).

#### Frost damage index of rice

The FD is usually calculated by the diurnal minimum temperature. FD will occur when the diurnal minimum temperature of rice during the seedling stage or milk stage is lower than 0 °C in Liaoning Province.

### Six scenarios of combined agrometeorological disaster for rice

A CAD is defined as follows: in the process of crop production, two or more types of meteorological disasters occur concurrently or alternately, or one type of meteorological disaster occurs in two or more different growth periods, resulting in crop losses^28^. The scenarios for CAD occurrence were based on three types: (1) one disaster in two periods (OD) scenario, where the same disaster occurred at different stages of rice production; (2) the two disasters (TD) scenario, where two kinds of disasters occurred in rice production; and (3) the multiple disasters (MD) scenario, where more than two kinds of disasters occurred during rice production. The scenarios for CAD in the rice growing season in Liaoning Province are shown in Table 3.

**Table 3:** Occurrence scenarios for combined agrometeorological disaster (CAD) in the rice growing season in Liaoning Province.

Types	Scenario	Disasters	Occurrence description
One disaster in two periods (OD)	One Disaster in two periods -1 (OD-1)	Frost damage (FD)	Disasters occur simultaneously at seedling stage and milk stage
	One Disaster in two periods -2 (OD-2)	Sterile-type cold damage (SCD)	Disasters occur simultaneously at booting stage and flowering stage
Two kinds of disasters (TD)	Two kinds of Disasters-1 (TD-1)	Delayed cold damage (DCD) + Frost damage (FD)	Disasters occur at seedling stage or milk stage, and accumulated temperature is insufficient in the growing season
	Two kinds of Disasters -2 (TD-2)	Delayed cold damage (DCD) + Sterile-type cold damage (SCD)	Disasters occur at booting stage or flowering stage, and accumulated temperature is insufficient in the growing season
	Two kinds of Disasters -3 (TD-3)	Sterile-type cold damage (SCD) + Frost damage (FD)	Disasters occur at seedling stage or milk stage, and occur at booting stage or flowering stage
Multiple Disasters (MD)	Multiple Disasters -1 (MD-1)	Delayed cold damage (DCD) + Sterile-type cold damage (SCD + Frost damage (FD)	Three kinds of disasters occur simultaneously

### Three scenarios of single agrometeorological disaster for rice

The scenarios for SAD occurrence were as follows: (1) SAD-d, in which only DCD occurred during rice production; (2) SAD-s, in which SCD occurred at only one stage during rice production; and (3) SAD-f, in which FD occurred at only one stage during rice production.

### Data processing

The DCD, SCD and FD were calculated in Liaoning Province between 1961 and 2017. The occurrence of CAD in the six scenarios and the occurrence of SAD in the three scenarios were identified from the data for DCD, SCD and FD. In calculating CAD statistics, when there was a MD in a certain station in one year, the TD was not counted; when there was a TD, then the OD was not counted.

This paper analysed the temporal and spatial distribution of CAD in Liaoning Province. The value of the ratio between the number of stations recording agrometeorological disasters and total stations (IOC) indicated the occurrence range of CAD in the region in regard to temporal variation. The frequency (P, %), was the percentage of years with CAD out of the total years^27^. The occurrence range of CAD was divided into local, regional and large-scale categories; an IOC lower than 20% was a local CAD; an IOC from 20% to 50% was a regional CAD; and an IOC greater than 50% was a large-scale CAD.

### Annual reduction in agrometeorological yield of rice

On the basis of the per unit yield of rice from 1961 to 2017, the trend yield was fitted using a quadratic polynomial method^26^, and the rice yield was separated to calculate the yield reduction rate affected by agrometeorological disasters. The formulas (Eqs. 6, 7, 8) are:

$$\begin{gathered} Y_{{\text{t}}} = \, 0.{1247}x^{{2}} + { 13}.{8}0{3}x + { 15}0.{7}\hfill \\ Y_{{\text{w}}} = Y_{{\text{a}}} - Y_{{\text{t}}} \hfill \\ \Delta Y = Y_{{\text{w}}} /Y_{{\text{t}}} \hfill \\ \end{gathered}$$

where *Y*_t_ is the trend yield, *x* is the time series number (1, 2……), *Y*_w_ is the agrometeorological yield, *Y*_a_ is the actual yield, *∆Y* is the yield reduction rate.

## Results

### Temporal characteristics of combined agrometeorological disasters

**Figure 2:**
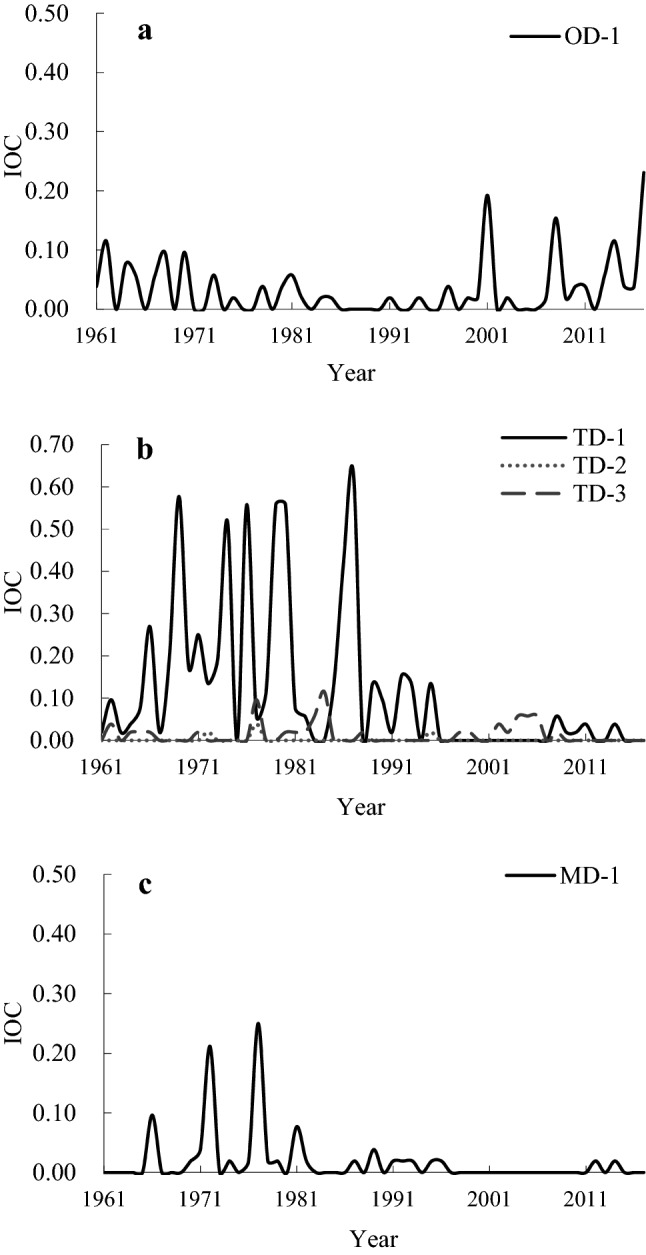
IOC (ratio between cold damage stations and total stations) for combined agrometeorological disasters (CAD) in different scenarios (**a** one disaster in two periods, OD; **b** two kinds of disasters, TD; **c** multiple disasters, MD).

The temporal variation of CAD was analysed in Liaoning Province from 1961 to 2017 using the IOC (Fig. 2). The maximum IOC for OD-1 was 0.231 occurring in 2017; there were 32 years with OD-1 at 1–12 stations. FD occurred at the seedling stage and the milk stage. There was no OD-2 in the past years (Fig. 2a).

TD-1 happened in 35 out of the past 57 years, when DCD and FD both occurred in the same rice growing season. The maximum IOC of TD-1 was 0.635 and it occurred in 1987. TD-2 happened in 3 years, when SCD occurred at the booting or flowering stage and DCD occurred during rice production. The maximum IOC of TD-2, a value of 0.038, occurred in 1977. A total of 20 years had TD-3 during rice production, with SCD in the booting or flowering stage, and FD in the seedling or milk stage. The maximum IOC of TD-3 was 0.115, in 1984 (Fig. 2b).

Between 1961 and 2017, the maximum IOC of MD-1 was 0.25, which happened in 1977. A total of 20 years showed evidence of the MD-1 scenario, when SCD, DCD and FD occurred simultaneously in the rice growing period (Fig. 2c).

From 1961 to 2017, TD-1 had the most years of occurrence among the six scenarios, followed by OD-1. TD-1 was the most widely distributed of the six scenarios, and TD-2 was the least frequent and least widespread. There was no occurrence of OD-2. In the past 57 years, the IOC values of the four scenarios have generally shown a downward trend, apart from the OD-1 scenario which showed an upward trend. From 1961 to 2000, TD-1 was the most widely distributed among the six scenarios; the distributions of most scenarios except OD-1 were smaller from 2001 to 2017.

Only TD-1 occurred over a large-scale grade in the six scenarios. There was one year at a regional level for OD-1 and 3 years for TD-1. Regionally, MD-1 occurred in 1972 and 1977 (Table 4). The scale of CAD was local in other years.

**Table 4:** Occurrence years of large-scale and regional combined agrometeorological disaster (CAD) in Liaoning Province.

Scenario	Large-scale grade	Regional grade
OD-1	–	2017
TD-1	1969, 1974, 1976, 1979, 1980, 1987	1966, 1971, 1986
TD-2	–	–
TD-3	–	–
MD-1	–	1972, 1977

### Spatial distribution of combined agrometeorological disasters

**Figure 3:**
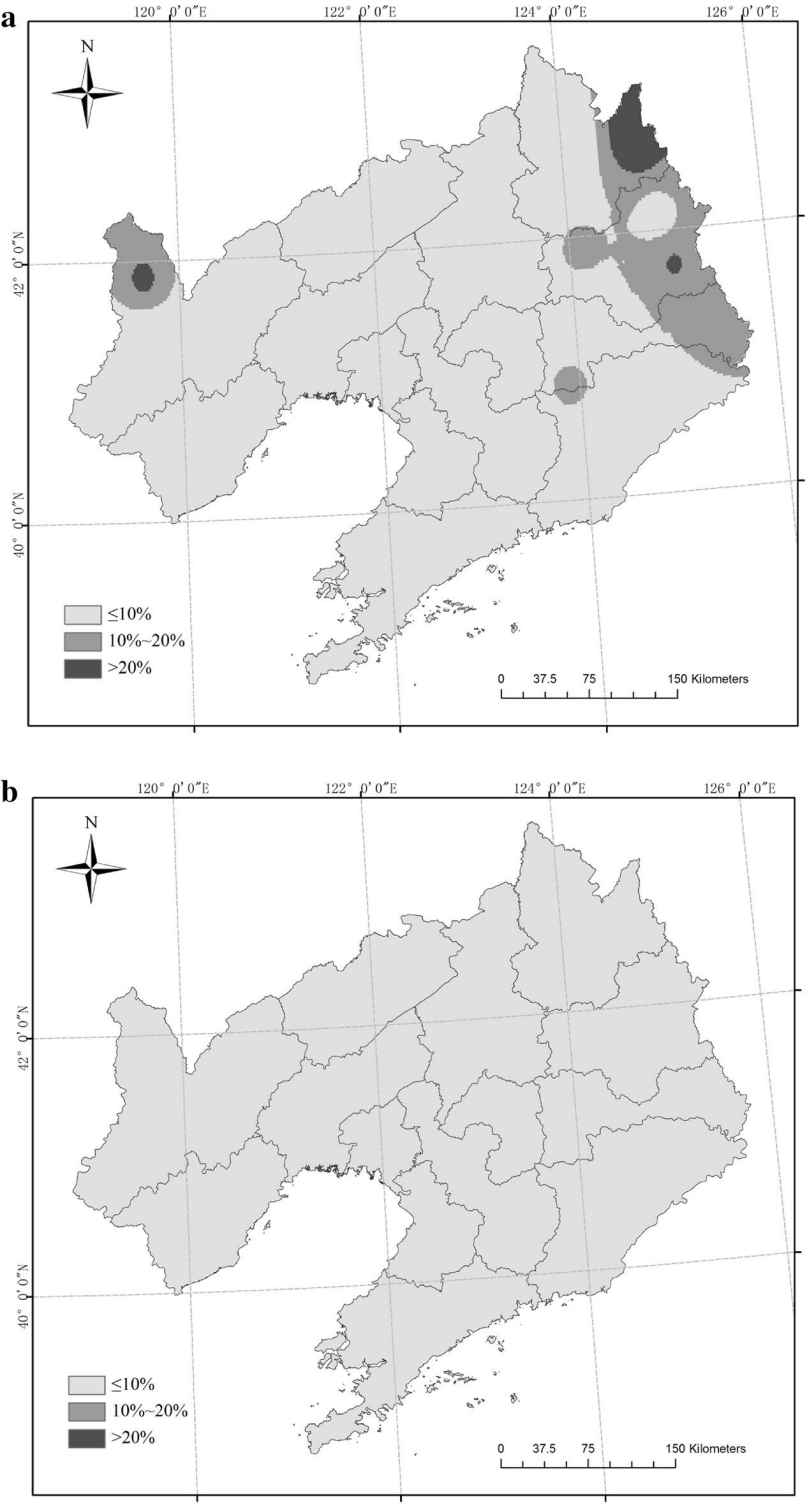

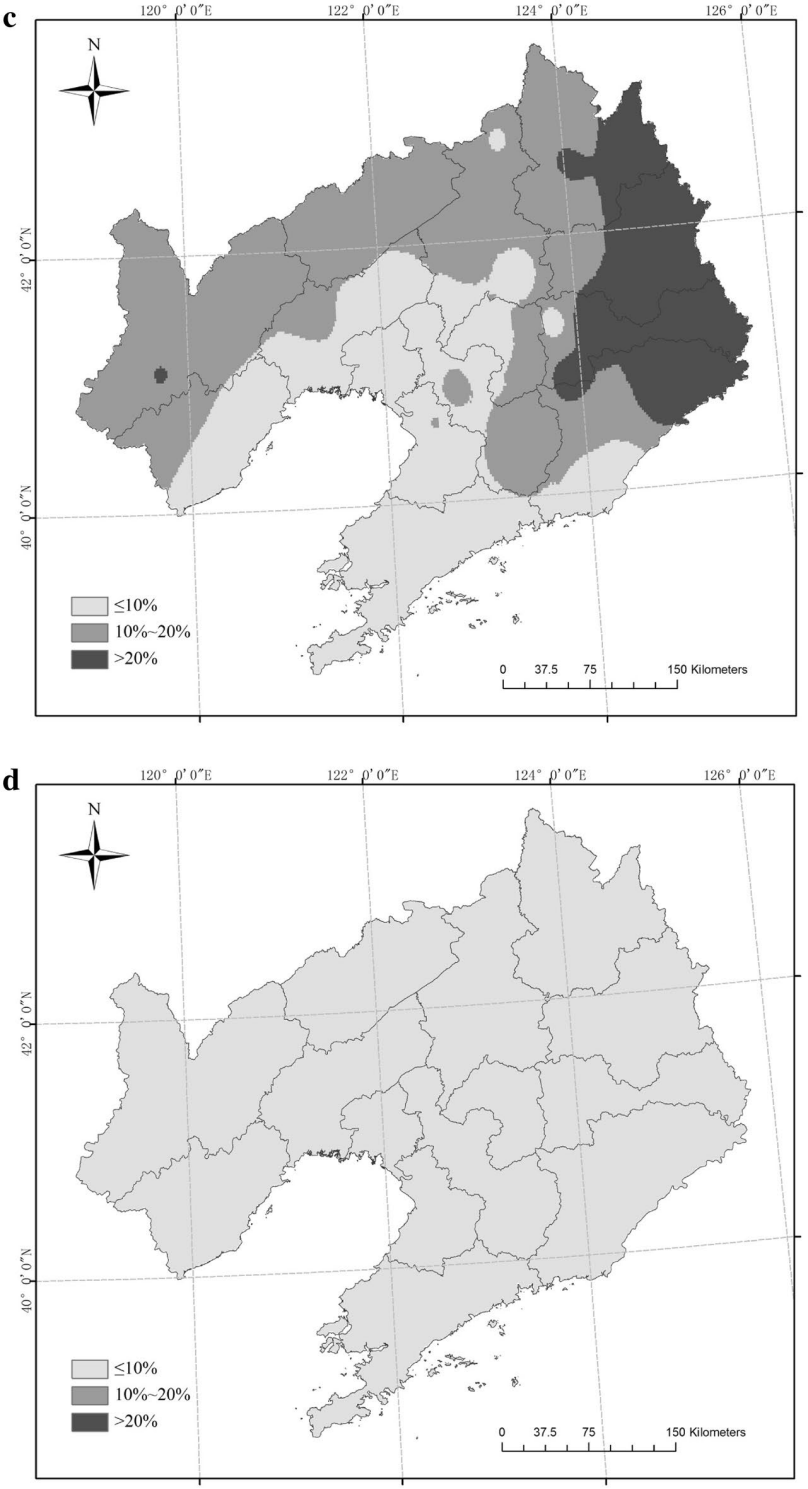

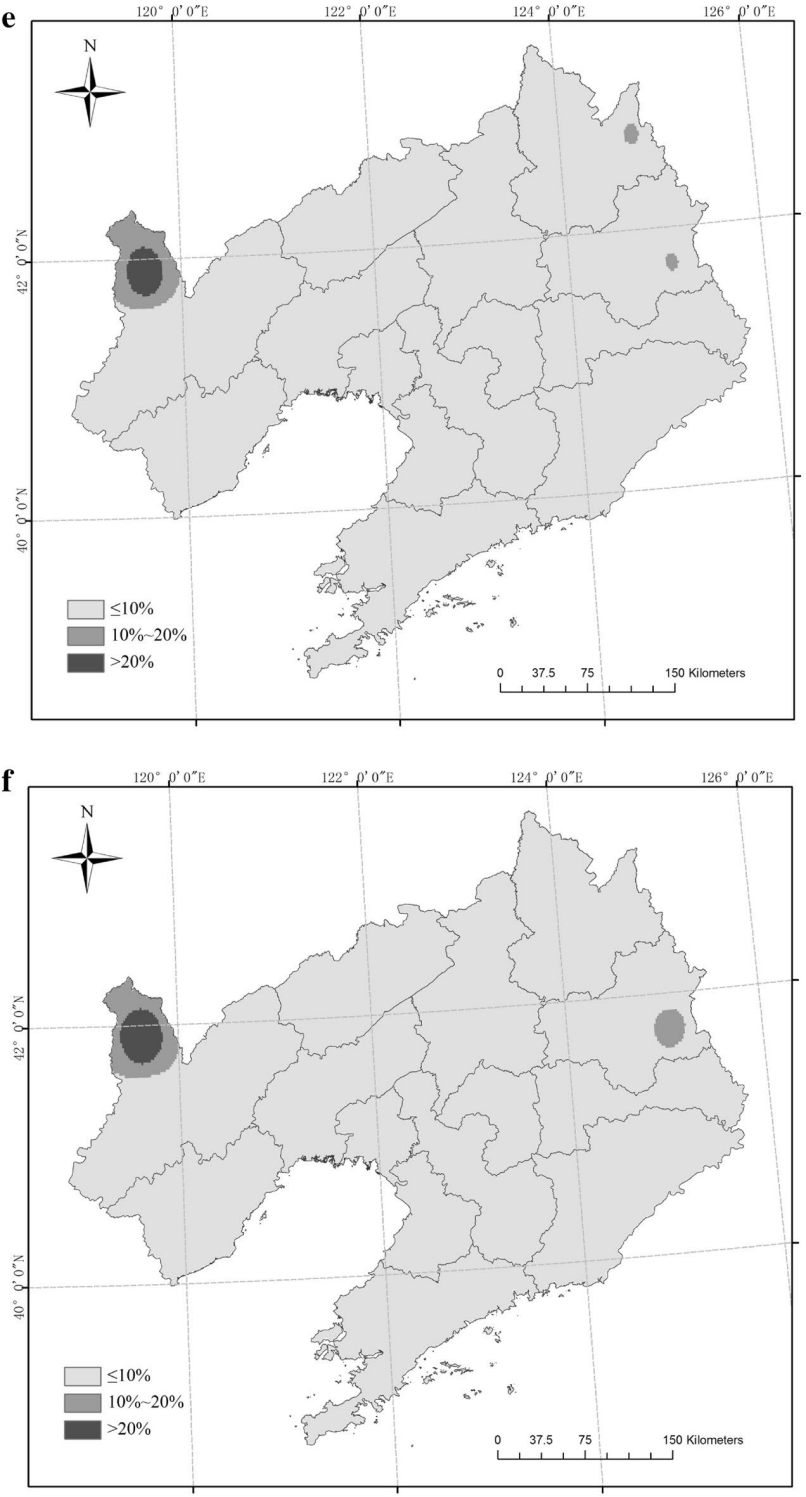
Frequency of combined agrometeorological disasters (CAD) in different scenarios (**a** OD-1, **b** OD-2, **c** TD-1, **d** TD-2, **e** TD-3, **f** MD-1). Maps generated in ArcGIS 9.3.

The six scenarios for rice CAD were mainly located in the northwest and northeast of Liaoning Province from 1961 to 2017 (Fig. 3). Three sites (Xifeng, Jianping, Xinbin) had an OD-1 frequency greater than 20% and three sites (Fushn, Huanren, Caohekou) had an OD-1 frequency ranging from 10% to 20%; the frequency at the other 46 sites was low (P ≤ 10%). The frequency of OD-2 was always less than 10%. For TD-1, nine sites had a frequency higher than 20%, and these were mainly distributed in northwest and east Liaoning Province. Eighteen sites had a frequency between 10% and 20% in northwest, central and east Liaoning; the frequency of the other 25 sites was low (P ≤ 10%). For TD-2, no site had a frequency greater than 10%. For TD-3, one site (Jianping) had a frequency greater than 20%, two sites (Xifeng, Xinbin) had a frequency ranging from 10% to 20%, and 49 sites had a frequency under 10%. One site (Jianping) had and MD-1 frequency greater than 20% and one site (Xinbin) had an MD-1 frequency between 10% and 20%.

## Discussion

### Characteristics of the single agrometeorological disaster scenarios

**Figure 4:**
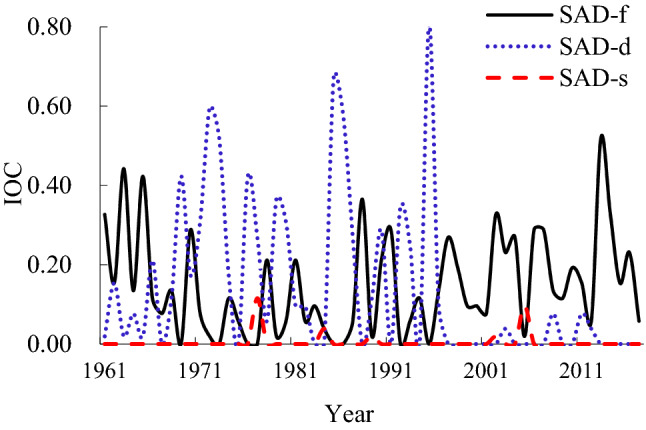
IOC change curve for single agrometeorological disasters (SAD) in different scenarios.

SAD-f occurred in 49 out of 57 years at different spatial scales, with a maximum IOC of 0.519 in 2013; SAD-d occurred in 33 years with a maximum IOC value of 0.808 in 1995; SAD-s occurred in 5 years, with a maximum IOC value of 0.115 in 1977 (Fig. 4). SAD-d showed a declining trend over the past 57 years, but the SAD-d frequency was higher than SAD-f and SAD-d. Since the mid-1980s, the frequency of SAD-f has increased, while the frequency and scale of SAD-s were relatively small.

A large-scale grade SAD-f event occurred in 2013 in Liaoning Province, and regional SAD-f occurred in 17 years. Five years showed a large-scale SAD-d and 11 years had regional SAD-d. There were no large-scale and regional years for SAD-s (Table 5).

**Table 5:** Occurrence years of large-scale and regional single agrometeorological disaster (SAD) in Liaoning Province.

Scenario	Large-scale grade	Regional grade
SAD-f	2013	1961, 1963, 1965, 1970, 1978, 1981, 1988, 1990, 1991, 1997, 2002, 2003, 2004, 2006, 2007, 2014, 2016
SAD-d	1972, 1973, 1985, 1986, 1995	1966, 1969, 1971, 1976, 1977, 1979, 1980, 1987, 1990, 1992, 1993
SAD-s	–	–

**Figure 5:**
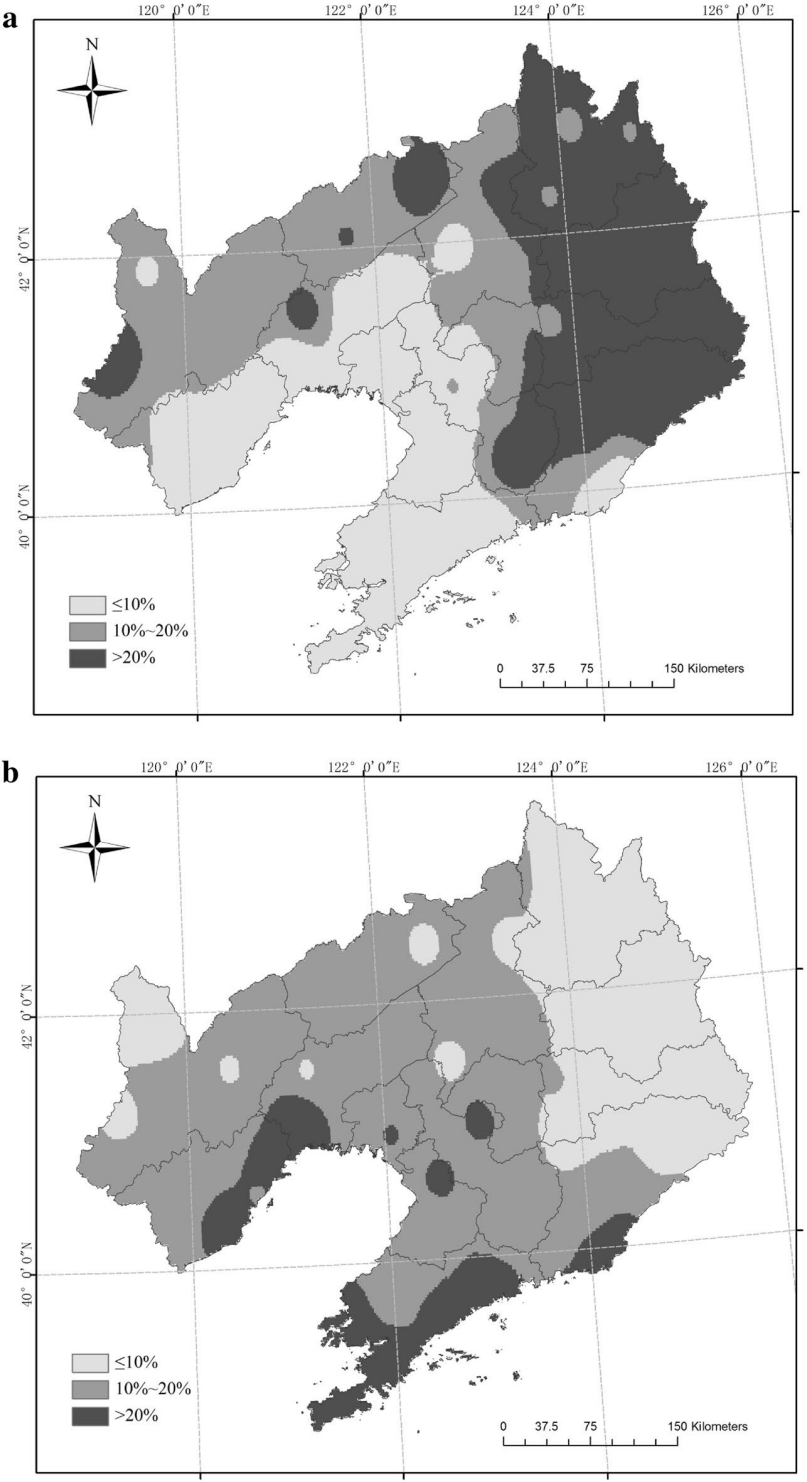

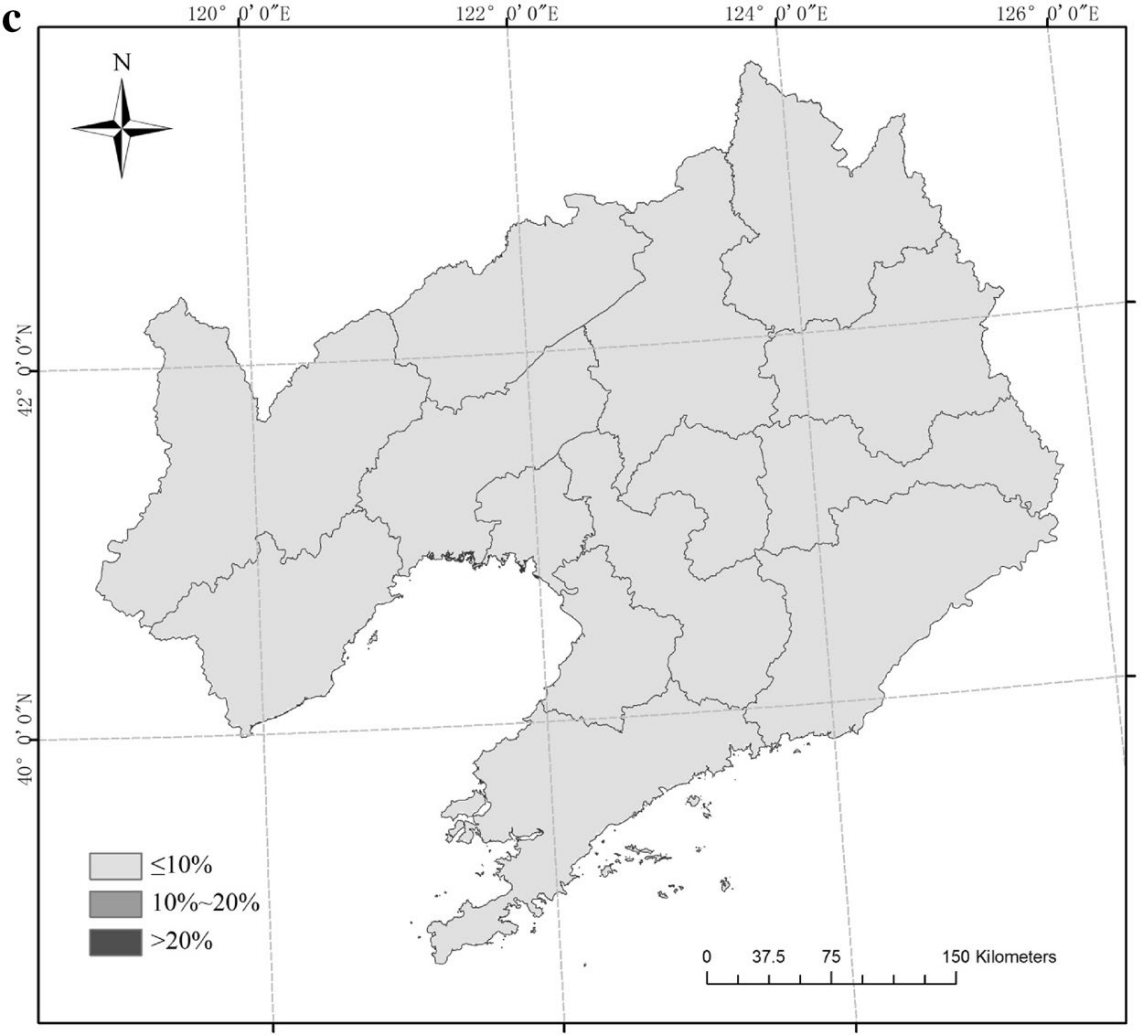
Frequency of single agrometeorological disasters (SAD) in different scenarios (**a** SAD-f, **b** SAD-d, **c** SAD-s). Maps generated in ArcGIS 9.3.

The occurrence of SAD-f was recorded at 15 sites with a frequency greater than 20%, 13 sites with frequency in the 10%–20% range, and 24 sites with a low frequency (P ≤ 10%) in Liaoning Province from 1961 to 2017 (Fig. 5). SAD-d occurred at 12 sites with a frequency higher than 20%, 22 sites with frequency between 10% and 20%, and 18 sites with a low frequency (P ≤ 10%). In the three scenarios, the occurrence frequency and distribution of SAD-f was the highest and SAD-s was the lowest.

### Comparison of the characteristics of single agrometeorological disasters and combined agrometeorological disasters

**Figure 6:**
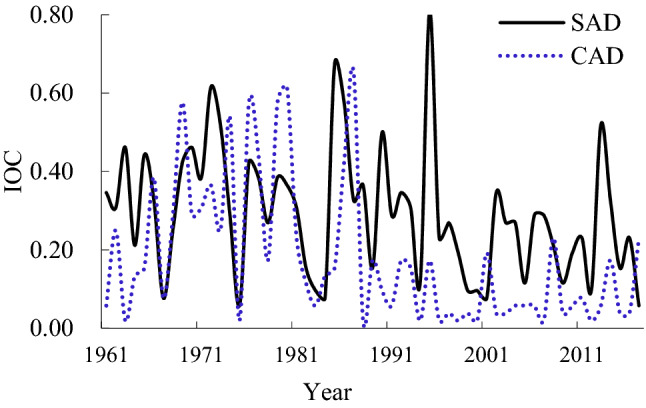
Change in the IOC for agrometeorological disasters in rice crops.

The maximum IOC of SAD was 0.808 in 1995 and the mean value was 0.294 for the 57 years of the study; the maximum IOC of CAD was 0.654 in 1987, and the mean value was 0.180 over the past 57 years; SAD and CAD occurred in all 57 years (Fig. 6). Both SAD and CAD showed declining trends from 1961 to 2017. The IOC was lower for CAD than for SAD for 42 years and higher than SAD for 14 years.

**Figure 7:**
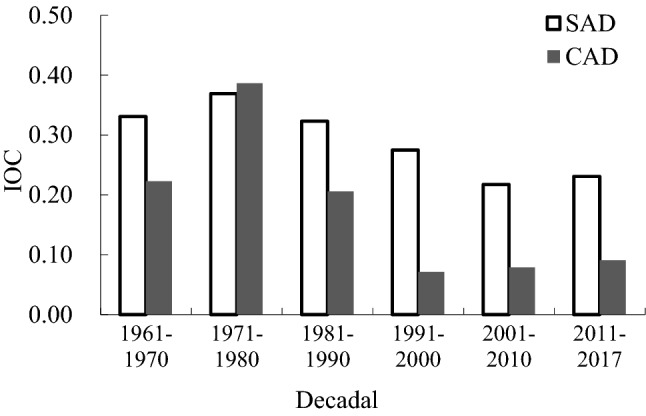
Interdecadal mean value of IOC for agrometeorological disasters in rice crops.

This paper analysed the mean IOC of SAD and CAD over six decades and found that the interdecadal mean value of the IOC in CAD was lower than that of SAD over five of the periods, but the IOC of SAD was lower than that of CAD in 1971–1980 (Fig. 7). The IOC of SAD showed a decreasing trend from the 1970s to the 2010s but showed an increasing trend after 2011. The IOC of CAD showed a decreasing trend from the 1970s to the 2000s, but showed an increasing trend after 2001 (Fig. 7).

**Figure 8:**
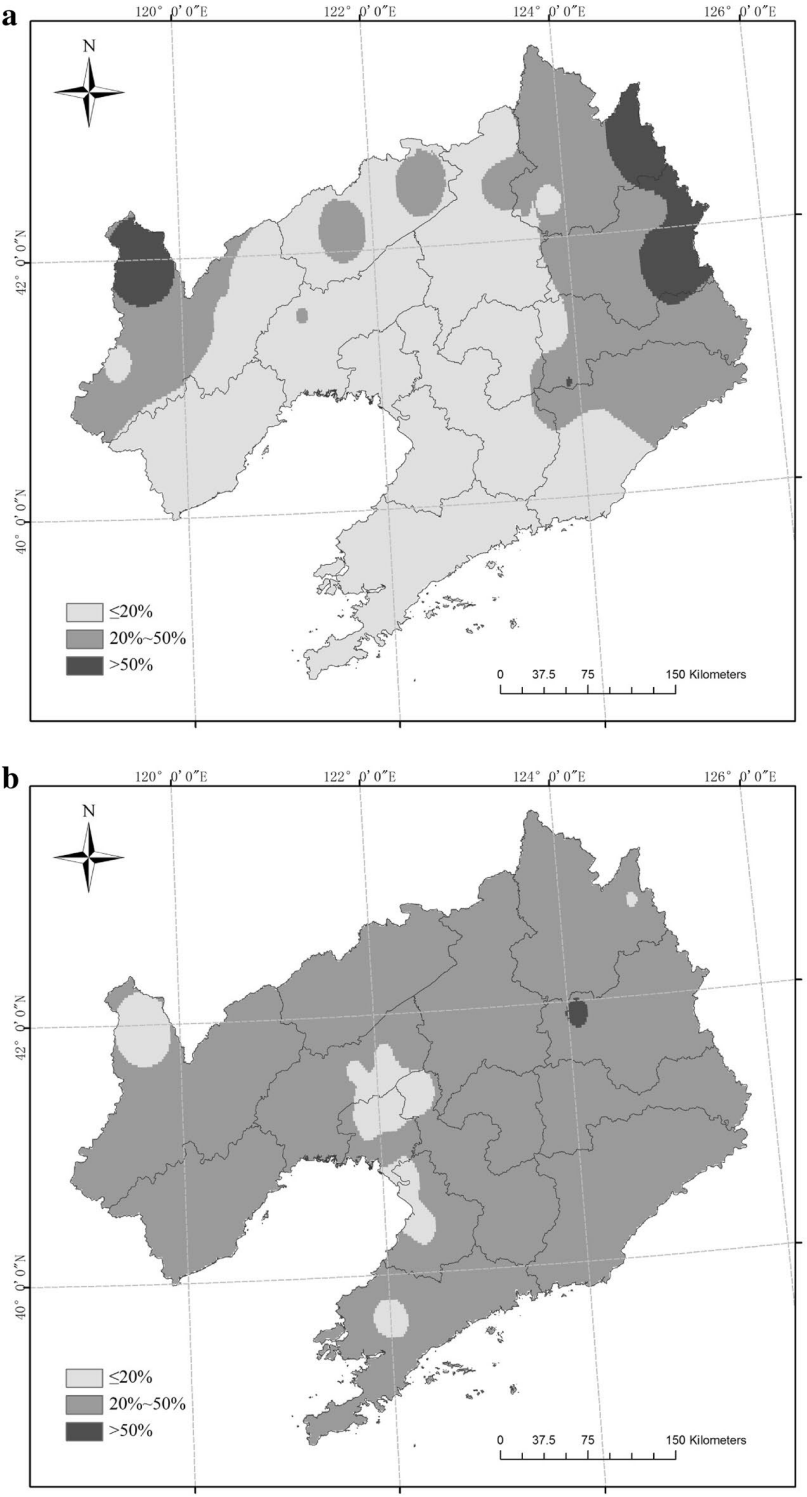
Frequency of agrometeorological disaster in rice crops (**a** combined agrometeorological disaster (CAD), **b** single agrometeorological disaster (SAD)). Maps generated in ArcGIS 9.3.

There was one site (Fushun) with a SAD frequency of more than 50% in Liaoning Province from 1961 to 2017, 42 sites with a frequency between 20% and 50%, and nine sites with a frequency lower than 20%. There were four sites (Xinfeng, Jianping, Xinbin and Caohekou) with a CAD frequency higher than 50%, 13 sites with a frequency in the range 20%–50%, and 35 sites with a frequency lower than 20% (Fig. 8). The frequency and range of CAD were less than those of SAD.

There has been little research into the temporal or spatial distribution of CAD for rice and its occurrence characteristics: most research has been on SAD. For example, studies have examined the characteristics of SCD, DCD, FD for rice in northeast China^26, 27, 29^, and the risk of multiple disasters for rice in northeast China^30, 31^. Han et al.^31^ analysed the risk of disaster using the reduction rate of rice yield in Liaoning Province from 1980 to 2011, and found that the high-risk areas were distributed in the west and northeast of Liaoning Province; higher rates of yield reduction in lean years were mainly found in western Liaoning and its surrounding areas. In this study, a higher frequency of CAD was mainly distributed in the northwest of Liaoning Province, while that of SAD occurred in the northeast of Liaoning Province. The median frequency of CAD occurred in the northwest and northeast of Liaoning Province, while that of SAD covered most areas in Liaoning Province. The range of medium and higher frequency occurrence in CAD was consistent with the distribution of high-risk and high yield reduction areas in the study of Han et al.^31^. Therefore, it can be speculated that the CAD scenarios might magnify the effect of each single disaster, and, therefore, CAD would more easily lead to a higher reduction in the rice yield.

### Comparison of the occurrence of single agrometeorological disasters and combined agrometeorological disasters

During the rice growing season in Liaoning Province, there were three scenarios of SAD and six scenarios of CAD. Compared with SAD, CAD had more scenarios and more complex processes, and its effect on rice was more difficult to evaluate. In SAD, the occurrence frequency and distribution of SAD-f and SAD-d were both high, when FD and DCD occurred alone in only one rice growth stage. In CAD, the occurrence frequency and distribution of TD-1, when FD and DCD occurred simultaneously, was the highest in the six scenarios. A single or combined occurrence of FD and DCD was most common disaster for rice in Liaoning Province. The occurrence frequency and distribution of OD-1 were both smaller than that of SAD-f, indicating that the occurrence was lower when FD happened at both the seedling and milk stages. SAD-s and OD-2 had the lowest frequency and range in all scenarios, indicating that DSD rarely appeared in SAD and CAD. The occurrence of SCD was not major disaster in the growth and development of rice in Liaoning Province, but the occurrence of DCD or FD, or both, was.

In this study, the occurrence frequency and range of SAD and CAD for rice showed declining trends in most sites over the past 57 years, which was consistent with the results of other studies. Studies on rice DCD and SCD concluded that cold damage events of rice in most areas of northeast China showed decreasing trends^26, 27^. Because of events such as climate warming, earlier warming in spring, delaying first frost dates and fewer low temperature days in summer, the trend of disasters was lower in rice planting areas^30^. However, although rice disasters showed a decreasing trend, local disasters may increase because of the frequent occurrence of climate anomalies. SAD-f and OD-1 scenarios in this study showed no significant decreasing trend, and even a partial increasing trend. Jiang et al.^29^ believed that the possibility of frequent SCD in north-east China was still high. According to Xi et al.^32^, cold periods would still occur in the growing season of rice in northeast China. Hu et al.^33^ concluded that the increase of SCD in northeast China was mainly because of the increase of climate variability, and most of the sites with increases were located in areas with decreasing temperature or no obvious trend of temperature increase.

Rice is a higher temperature-loving crop, which is mainly restricted by temperature conditions during its growing season. Liaoning Province is in the south of the rice planting area of the colder regions in China. Because of the relatively low latitude, heat conditions during the rice growing season were better than those in Jilin and Heilongjiang to the north of Liaoning Province. The climatic risk of cold damage in the rice growing season was lower than other regions in northeast China^34^. The occurrence of CAD was generally caused by low temperatures, which were the dominant factor. When two or more disasters occur together, there is a coupling or amplifying effect on rice growth compared with a single disaster.

A comparison of the rice yield reduction rates in the years when CAD or SAD occurred in more than 50% of stations in Liaoning Province revealed that the former happened in 5 years, 1969, 1974, 1976, 1980 and 1987, whereas the latter happened in 7 years, 1972, 1973, 1985, 1986, 1990, 1995 and 2013. When CAD was the major occurrence, the average yield reduction rate in the five years was 10.6%. The yield reduction rate in 1969 was 34.6%, which was the highest in the past 57 years. When SAD was the major occurrence, the average yield reduction rate in the seven years was 9.8%. The average yield reduction rate in the years when CAD dominated was greater than in the years when SAD dominated. Therefore, it can be speculated that CAD has a greater effect on rice growing than any single disaster within CAD. However, it is difficult to quantify the effect on rice yield of CAD, and further controlled field experiments should be conducted to verify these. It is difficult to control field experiments that are limited by conditions and facilities.

### Comparison of the occurrence of agrometeorological disasters in years having rice yield reductions

On the basis of the rice yield reduction rate in calculations Liaoning Province from 1961 to 2017, a total of 10 years (Table 6) were screened. Six years had large-scale disasters (including SAD and CAD) and four years had regional disasters. In 1969, which showed the highest yield reduction rate (34.6%), 30 sites had TD-1 disasters and the other 22 sites had SAD-f disasters. In 1972, the second highest reduction year (29.1%), 11 sites had MD-1 disasters, i.e. three kinds of disasters occurred, seven sites had TD-1 disasters, one site had a TD-2 disaster, 31 sites had SAD-d disasters, one site had a SAD-f disaster, and only one station had no disaster. The TD-1 disaster, i.e. delayed cold damage and frost injury, was the most frequent CAD over the years, and SAD-d, i.e., delayed cold damage, was the most frequent SAD. The occurrence of single and combined agrometeorological disasters in different regions strongly affected the rice yield. Generally, the larger the disaster range, the higher the yield reduction. However, some years were not completely consistent with this conclusion. The yield reduction rate was also related to the type, severity, occurrence period and geographical location of the disasters.

**Table 6:** Comparison of agrometeorological disasters in years having greater than 10% rice yield reduction rates in Liaoning Province.

Year	Yield reduction rate (%)	IOC for all disasters	Station numbers in CAD	Station numbers in SAD	CAD types (station numbers)	SAD types (station numbers)
1961	23.5	0.40	3	18	OD-1(2), TD-1(1)	SAD-f (17), SAD-d (1)
1962	21.8	0.56	13	16	OD-1(6), TD-1(5), TD-3(2)	SAD-f (8), SAD-d (8)
1969	34.6	1	30	22	TD-1(30)	SAD-d (22)
1972	29.1	0.98	19	32	TD-1(7), TD-2(1), MD-1(11)	SAD-f (1), SAD-d (31)
1976	15.6	1	30	22	TD-1(29), MD-1(1)	SAD-d (22)
1985	12.6	0.83	8	35	OD-1(1), TD-1(7)	SAD-d (35)
1989	22.0	0.33	9	8	TD-1(7), MD-1(2)	SAD-f (1), SAD-d (6), SAD-s (1)
1995	25.1	0.98	9	42	TD-1(7), TD-2(1), MD-1(1)	SAD-d (42)
2006	12.7	0.35	3	15	TD-3(3)	SAD-f (15)
2010	14.8	0.25	3	10	OD-1(2), TD-1(1)	SAD-f (10)

*IOC:* ratio of the number of stations recording agrometeorological disasters to total stations, *CAD:* combined agrometeorological disasters, *SAD:* single agrometeorological disaster.

**Figure 9:**
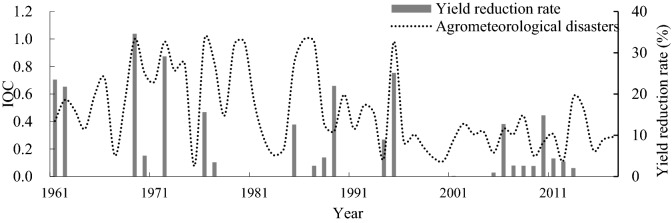
The IOC change curve of all agrometeorological disasters and the rice yield reduction rate from 1961 to 2017.

**Figure 10:**
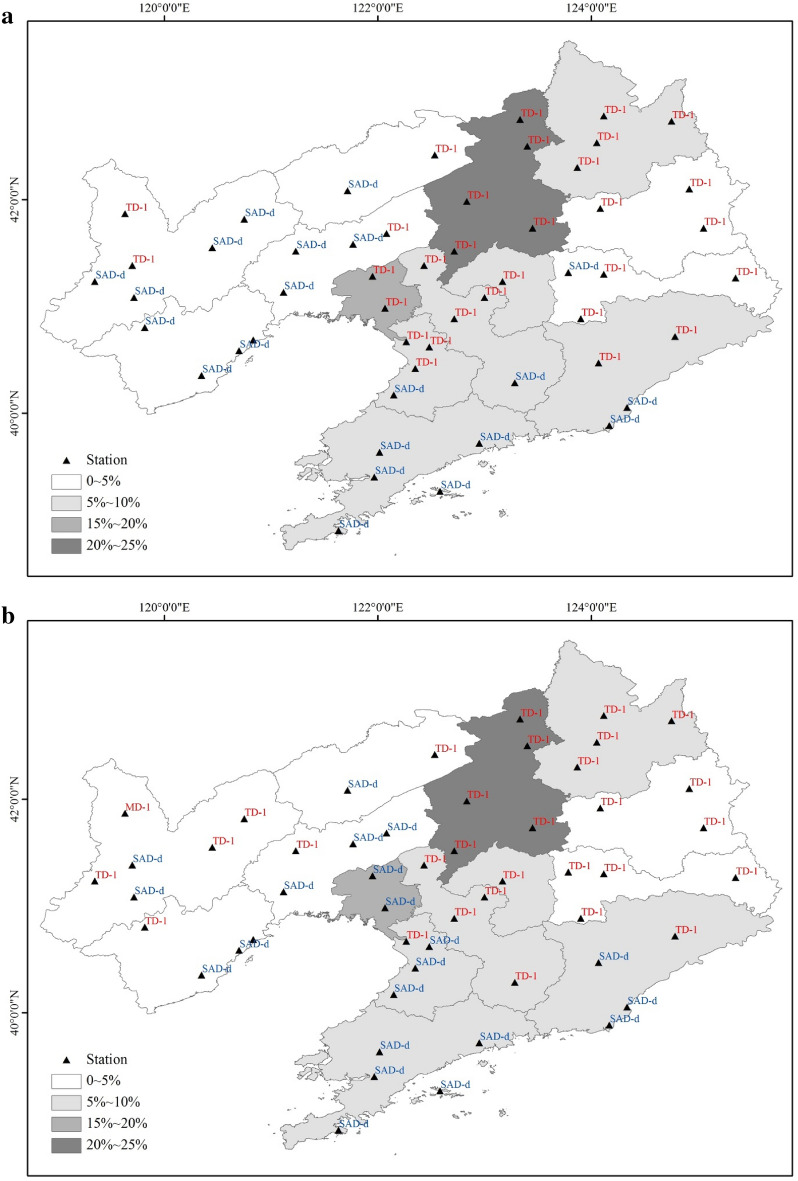
Distributions of the types of agrometeorological disasters and the percentages of rice planting areas in different regions of Liaoning Province in 1969 and 1976 (**a**: 1969; **b**: 1976). Maps generated in ArcGIS 9.3.

In every year from 1961 to 2017, CAD or SAD occurred in Liaoning Province, and the rice yields declined in 23 of the 57 years owing to meteorological disasters (Fig. 9). Although meteorological disasters occurred in the other 34 years, there was no reduction in rice production, which may be related to the gradient of the disaster or the spatial distribution of the rice planting areas. The rice yield reduction rates in 1969 and 1976 were 34.6% and 15.6%, respectively. In these two years, CAD occurred at 30 stations and SAD occurred at 22 stations, and TD-1 was the main type of CAD, whereas SAD-d was the main type of SAD. Using statistical data, on the rice planting area of each city in Liaoning Province, the provincial area can be divided into four regions. The first region was Shenyang City, which has the largest rice planting area, accounting for 20%–25% of the total rice planting area; the second region was Panjin City, accounting for 15%–20% of the total rice area; the third region encompassed Tieling and other six cities, accounting for nearly 50% of the total rice area, with each city representing 5%–10%; and the fourth region encompassed Jinzhou and five other cities, accounting for 10%–15% of the total, with each city representing 0–5%. As shown in Fig. 10a,b, TD-1 occurred in the first region in both 1969 and 1976 and in the second region in 1969. SAD-d occurred in the second region in 1976. In the third region, TD-1 occurred at more stations of 1969 than in 1976. The rice area in the first three regions accounted for nearly 80% of the total rice area, and CAD occurred more often than SAD in these regions. Thus, there was a greater yield reduction rate in 1969 than in 1976.

The occurrence characteristics of single disasters or the risk of yield reduction were analysed in previous studies, but the quantitative effect on rice production was rarely evaluated. Ji et al.^26^ reported that the delayed cold damage in 1961, 1962, 1969, 1972, 1976, 1989 and 1995 was so severe that there was a large reduction in rice production. In our paper, we examined the occurrence of not just one disaster, i.e. delayed cold damage, over time, but also other types of disasters including SAD and CAD. For example, in 1972 and 1976, the disaster scenario affecting the largest number of stations was TD-1, i.e., both delayed cold damage and frost damage occurred in the growing season of rice. In 1961, the most widespread damage came from a single disaster—frost damage. According to the records^35^, Liaoning Province experienced frost damage in 1961, 1962, 1969, 1972, 1976 and 1995, and the rice yield was seriously reduced. Most regions of Liaoning Province experienced both delayed cold damage and frost damage in 1976 and 1995. There was a low temperature during the critical period of rice growth (mid-July to mid-August) in 1995. In 1985, the growing season in most areas was characterized by unusually persistent low temperature and little sunshine. These statistics were basically consistent with the conclusion of this study. In the process of rice production, a variety of disasters occurred caused by low temperature, such as delayed cold damage, frost damage and sterile cold damage.

## Conclusions

There were six possible scenarios of CAD in the rice growing season in Liaoning Province from 1961 to 2017, with OD-1, OD-2, TD-1, TD-2, TD-3 and MD-1 occurring in 32, 0, 35, 3, 20 and 20 years, respectively. Large-scale TD-1 only appeared for 6 years, while regional OD-1, TD-1, TD-2 and MD-1 appeared for 1, 3 and 2 years, respectively. The six scenarios were mainly found in the northwest and northeast of Liaoning Province. OD-1 with a frequency greater than 20% occurred at three sites and TD-1 at nine sites. Three sites of OD-1 and 18 sites of TD-1 showed a disaster occurrence with a frequency in the 10%–20% range. The occurrence frequency of other disasters was less than 10%. The IOC values in the four most common scenarios showed declining trends but the OD-1 scenario showed an upward trend. The distributions and frequency of TD-1 were the highest in the six scenarios.

In the past 57 years, the occurrence of CAD showed a significant downward trend, and the decline rate was more rapid than that of SAD. CAD and SAD occurred in all 57 years. There were 42 years with an IOC value in CAD lower than that of SAD, and 14 years where it was higher than SAD. Four sites in CAD and one site in SAD had a frequency greater than 50%. There were 13 sites with a frequency between 20% and 50% in CAD, and 42 sites in SAD. Compared with SAD, the occurrence range and frequency of CAD was lower, and the interdecadal mean value of the IOC in CAD was lower than that of SAD in all decades except 1971–1980. High frequencies of SAD were mainly found in the northeast of Liaoning Province, while those of CAD were mainly distributed in the northwest. The major agrometeorological disasters affecting rice production in Liaoning Province were DCD, FD or a combination of the two.

The occurrence frequency and range of SAD and CAD events showed decreased trends in rice cropping areas in the past 57 years. Under the background of climate change, early spring warming, delays in the first frost date and decreases in low temperature events in summer were the main reasons for the decrease of cold disaster events in rice. However, because of the frequent occurrence of climate anomalies, extreme cold events still exist. Disasters in SAD-f and OD-1 showed no significant decreasing trend, and even slightly increased. Therefore, both SAD and CAD were still high probability events in the rice growing areas of Liaoning Province. Further study is needed on the causes and characteristics of CAD occurrences in relation to the climate background and dominant weather types because of their complexity.

Compared with SAD, CAD had more scenario types and more complex occurrence processes. Under the CAD scenarios, the effects of different disasters may be magnified, making reductions in rice yields more likely. CAD or SAD occurred in every year from 1961 to 2017, and the rice yields declined in 23 of the 57 years. A comparison of the rice yield reduction rates in the years when CAD or SAD occurred in more than 50% of stations in Liaoning Province revealed a greater average yield reduction rate in former than in latter. Nearly 80% of the total rice area in 1969 had a yield reduction rate greater than 10%, and CAD occurred more often in 1969 than in 1976 in this region, which led to a greater yield reduction rate in 1969 than in 1976. Meteorological disasters occurred in 34 years that showed no reduction in rice production, and this may be related to the gradient of the disaster or the spatial distribution of the rice planting areas. Further controlled field experiments should be conducted to quantify the effects of CAD on rice yield.
